# The Medical and Surgical Register of the United States

**Published:** 1892-09

**Authors:** 


THE MEDICAL AND SURGICAL REGISTER OF THE UNITED STATES.—R. L. Polk & Co., Detroit, Mich., have now in course of compilation the third edition of this valuable publication. The Medical and Surgical Register has become a standard work and occupies its field exclusively; it gives a complete record of the physicians of the United States, colleges and date of graduation, besides much other valuable information of interest to the profession. It is the only work that has ever made the attempt to record the physicians of the United States according to the medical college training each individual has received, and is remarkably free from the mistakes inseparably connected with the preparation of such a volume. The work is worthy the support of the profession, and we trust that every physician will respond promptly to the request of the publishers for data which will aid materially the compilation of the work.

